# Cytotoxicity of Hybrid Noble Metal-Polymer Composites

**DOI:** 10.1155/2022/1487024

**Published:** 2022-10-11

**Authors:** Anton Tkachenko, Pavlo Virych, Valeriy Myasoyedov, Volodymyr Prokopiuk, Anatolii Onishchenko, Dmytro Butov, Yuliia Kuziv, Oleg Yeshchenko, Sican Zhong, Guochao Nie, Nataliya Kutsevol

**Affiliations:** ^1^Research Institute of Experimental and Clinical Medicine, Kharkiv National Medical University, Kharkiv 61022, Ukraine; ^2^Department of Biochemistry, Kharkiv National Medical University, Kharkiv 61022, Ukraine; ^3^Faculty of Chemistry, Taras Shevchenko National University of Kyiv, Kyiv 01033, Ukraine; ^4^Department of Medical Biology, Kharkiv National Medical University, Kharkiv 61022, Ukraine; ^5^Department of Cryobiochemistry, Institute for Problems of Cryobiology and Cryomedicineof the National Academy of Sciences of Ukraine, Kharkiv 61015, Ukraine; ^6^Department of Phthisiology and Pulmonology, Kharkiv National Medical University, Kharkiv 61022, Ukraine; ^7^Faculty of Physic, Taras Shevchenko National University of Kyiv, Kyiv 01033, Ukraine; ^8^Guangxi Key Laboratory of Agricultural Resources Chemistry and Biotechnology, Yulin Normal University, Yulin 537000, China

## Abstract

The aim of the present research was to assess the cytotoxicity of gold and silver nanoparticles synthesized into dextran-graft-polyacrylamide (D-PAA) polymer nanocarrier, which were used as a basis for further preparation of multicomponent nanocomposites revealed high efficacy for antitumor therapy. The evaluation of the influence of Me-polymer systems on the viability and metabolic activity of fibroblasts and eryptosis elucidating the mechanisms of the proeryptotic effects has been done in the current research. The nanocomposites investigated in this study did not reduce the survival of fibroblasts even at the highest used concentration. Our findings suggest that hybrid Ag/D-PAA composite activated eryptosis via ROS- and Ca^2+^-mediated pathways at the low concentration, in contrast to other studied materials. Thus, the cytotoxicity of Ag/D-PAA composite against erythrocytes was more pronounced compared with D-PAA and hybrid Au/polymer composite. Eryptosis is a more sensitive tool for assessing the biocompatibility of nanomaterials compared with fibroblast viability assays.

## 1. Introduction

Noble element-based nanoparticles are promising bioengineering materials that can be used for diagnostic and therapeutic purposes. In particular, there is accumulating evidence that gold and silver nanoparticles can find the potential application in cancer diagnostics and treatment, bioimaging, biosensing, drug delivery, and treatment of infectious diseases as antibacterial, antifungal, and antiviral agents [1, 2]. Unique favorable properties of nanobased noble metal nanoparticles can be attributed to an increase in the surface-to-volume ratio and surface plasmon resonance, electronic, photothermal, and catalytic properties [2, 3]. The relatively high surface area of nanoparticles facilitates their uptake by cells and increases the biological activity in this way [4]. In addition, noble metal-based nanoparticles are characterized by high retention and, thus, accumulation in tumor cells providing target action [5].

The field of noble metal nanotechnology has been expanding for years to improve the stability of nanomaterials, their efficacy and biocompatibility, and to reduce the toxicity [6]. Another concern that prevents the translation of noble metal-based nanoparticles into practice is a limited elimination of them from the body, which increases their toxicity inducing damage to tissues and cells [7]. Moreover, the cytotoxicity of noble metal nanoparticles can be increased due to their instability and a trend to aggregate. This leads to the loss of unique nanosize-associated properties and reduction of efficacy [8]. However, the formation of metal/polymer nanocomposites is efficient to overcome this drawback, maintain the properties of nanosized materials, and reduce their toxicity. In these nanocomplexes, polymers stabilize the structure, act as a matrix, and protect metal nanoparticles [9].

It has been demonstrated that the toxicity of metal/polymer composites is associated primarily with metal components, i.e., fillers. In their turn, metals can exert toxic effects directly or they can be promoted by ion release [10]. It is worth mentioning that toxic effects of metal nanoparticles are mediated by oxidative stress induced by excessive reactive oxygen species (ROS) generation, mitochondrial dysfunction, activation of necrosis, apoptosis, autophagy, damage to cell membranes, and macromolecules such as proteins and DNA [11]. The use of biopolymeric carrier or polymer coatings is an efficient tool to modulate the stability and metal ion release, which contributes to the reduction of toxicity [12].

During the past few years, gold and silver nanoparticles (AuNPs and AgNPs) with their unique properties have led to new and exciting developments with enormous potential in biology and medicine [13, 14]. It has been reported that Au and Ag nanoparticles have become one of the most investigated and explored nanotechnology-derived nanostructures, given the fact that nanosilver and nanogold-based materials proved to have interesting, challenging, and promising characteristics suitable for various biomedical applications [15, 16]. Biomedical potential of AgNPs and AuNPs is oriented toward the therapeutically enhanced healthcare practice. Metal nanoparticles proved to have impressive potential for the development of novel antimicrobial agents, drug-delivery systems, detection and diagnosis platforms, biomaterial and medical device coatings, regeneration materials, and performance-enhanced therapeutic alternatives. Given the impressive biomedical-related potential applications of AuNPs and AgNPs, considerable efforts have been undertaken to understand the intricate mechanisms of their biological interactions and possible toxic effects.

Polymeric nanomaterials have become a prominent area of biomedical research. The application of biocompatible water-soluble polymers in nanomedicine can improve bioavailability, pharmacokinetics, and, therefore, the effectiveness of therapeutic agents [17, 18]. Polymers can be an efficient matrix for *in situ* synthesis of metal NPs preventing their aggregation and allowing regulating the nanoparticle size [19–21]. The multifunctionality of polymer molecules with unique chemical and biological properties ensures better delivery and a controlled release of various therapeutic agents. They are stable in the body, can provide optimal pharmacokinetics and biodistribution and ensure the protection of healthy tissues and accumulation of drugs in affected tissue.

The aim of this study was to evaluate the cytotoxicity of hybrid metal-polymer composites, synthesized in situ into polymer matrix dextran-graft-polyacrylamide, against normal erythrocytes and fibroblasts by estimating their ability to induce eryptosis with a focus on ROS- and Ca^2+^-mediated pathways and viability of fibroblasts.

## 2. Materials and Methods

### 2.1. Synthesis of Polymer Nanocarrier and Its Characteristics

To create nanosystems for biological study, star-shaped copolymers with dextran (D) core and grafted polyacrylamide chains (PAA) were used [22, 23]. The synthesis, characterization, and peculiarities of intramolecular structure of series of copolymers with dextran core and grafted PAA chains at the variation of the size of dextran core and amount of PAA-grafts were reported in [22, 23]. High efficiency of these copolymers as nanocarriers for drug delivery of anticancer drugs doxorubicin and cisplatin [24], as well as for synthesis and use the multicomponent nanosystems for the photodynamic therapy, was shown [25, 26]. In the present research, the copolymer with dextran core (Mw = 70000 g/mol) and 5 grafted PAA chains was used. This copolymer possesses low polydispersity (Mw/Mn), high molecular weight (Mw), and optimal molecular structure with mushroom conformation of grafts [22, 23] (Таble 1). This copolymer is further referred to as D-PAA.

#### 2.1.1. Synthesis of D-PAA Copolymers

Dextran (Sigma-Aldrich) with Mw = 70,000 g/mol was dissolved in deionized water, and argon was passed for 20 min. Then, the calculated amount of initiator was injected. Cerium (IV) ammonium nitrate (Aldrich) was an initiator of radical polymerization. The acrylamide (AA) monomer was added 20 min after the initiator. The monomer was prerecrystallized twice from chloroform. After the initiator injection, the oxidation-reduction reaction took place, and macroradicals on the dextran macromolecule were formed [22, 23]. The number of the grafts (*N*) varied by the dextran-to-initiator ratio (*N* = [molCe(IV)]/[molDextran]) and was equal to 5.

The polymerization was carried out for 24 hours. Upon completion of the polymerization reaction, the polymer was precipitated in acetone and freeze-dried.

The molecular parameters of the polymer sample determined by size exclusion chromatography are shown in Table 1.

### 2.2. In Situ Synthesis of Ag and Au Nanoparticles into D-PAA Matrix

The Ag and Au nanoparticles were synthesized in situ into polymer matrix D-PAA. The silver and gold nanoparticles were synthesized by reduction of the AgNO_3_ and HAuCl_4_ precursors using sodium borohydride (NaBH_4_) as reductant. All chemicals were purchased from Sigma-Aldrich. The synthesis of metal sols was carried out *in situ* into aqueous solution of dextran-graft-polyacrylamide copolymer.

Initially, 0.05 mL of 0.1 M precursors dissolved in bidistilled water was added to 1 mL of water polymer solution (*C* = 1 g∙L^−1^) and stirred for 20 min. Then, 0.1 mL of 0.1 M aqueous solution of NaBH_4_ was added. The final solution was stirred for 30 min. The appearance of ruby-red color indicated the formation of AuNPs, and the reddish-brown color pointed to the formation of AgNPs.

Characterization of these nanosystems by UV-vis, dynamic light scattering, SAXS, and TEM was reported in our recent works [19, 20, 27–29].

These nanosystems were used for preparation of multicomponent nanocomposites for chemo- and photodynamic anticancer therapy [18, 25, 29]. High photodynamic efficiency of multicomponent nanosystems containing AuNPs and photosensitizer (PS) chlorin e6 encapsulated into D-PAA nanocarrier simultaneously was demonstrated. AuNPs was reported to reveal a local heating process under irradiation of nanosystem Au/D-PAA with laser [30], which could provoke the effect of hyperthermia at PDT treatment.

It was shown [31] that polymer D-PAA loaded with cisplatin yielded a dose-dependent decrease in viability of chronic myelogenous leukemia and histiocytic lymphoma cells. The effect of nanosilver on cell viability was lower than that of polymer/cisplatin composite. The data from the cytotoxic studies indicate that nanosilver induces toxicity in cells. However, when the copolymers were conjugated to both nanosilver and cisplatin, such a nanosystem displayed less cytotoxic effect compared to the conjugates of dextran-graft-polyacrylamide and cisplatin.

The aim of current research was to study the cytotoxicity of AgNPs and AuNPs synthesized into D-PAA matrix as the basic nanosystems oriented toward the therapeutically enhanced healthcare practice, partially for chemo- and photodynamic anticancer therapy.

### 2.3. Animals

Six female adult WAG rats (age: 4-5 months; weight: up to 200 g) were used as blood donors for estimating eryptosis indices. Blood samples were collected in sterile K_2_EDTA vacutainers (Guangzhou, China). In addition, eight rat fetuses (gestation days 15-20) were used for obtaining skin fibroblasts [32].

The study was performed in conformity with the Directive 2010/63/EU for the Protection of Animals Used for Scientific Purposes and the European Convention for the Protection of Vertebrate Animals used for Experimental and other Scientific Purposes (EST 123). The approval from the Commission on Ethics and Bioethics (Kharkiv National Medical University, Kharkiv, Ukraine; minutes #3 dated August 28, 2020) was received.

### 2.4. Fibroblast Cultures and Incubation Conditions

To perform cell viability assays, we used dermal fibroblasts isolated from rat embryos by enzymatic tissue dissociation [32]. After isolation and fragmentation of skin, incubation with 0.25% trypsin-EDTA purchased from BioWest (France) for 1 h at 37°С was performed with a magnetic stirrer. After addition of 10% fetal bovine serum (FBS, Lonza, Germany), the cells were washed in Dulbecco's modified eagle medium (DMEM) purchased from BioWest (France). The cells were seeded in 25 cm^2^ SPL culture flasks (Republic of Korea). When the monolayer confluence reached 100%, the cells were harvested by 0.25% trypsin-EDTA. The fibroblasts used in the experiment underwent 3-4 passages.

The fibroblasts were incubated with a polymer (panel a), hybrid Ag-polymer composite (panel b), and hybrid Au-polymer composite (panel c) at concentrations of 0-0.5-1-2-5-10-20-30-50 mg/L for 24 h to assess the monolayers visually and perform cell viability tests.

Images of cell cultures were acquired using an inverted phase contrast microscope NIB-100 (Delta Optical, Poland) equipped with a UCMOS 3100 camera (SIGETA, Hangzhou, PRC). ToupView v.3.7 (Hangzhou Toup Tek Photonics Co. Ltd, Hangzhou, PRC) and ImageJ V.1.48. (National Institute of Health, USA) software were used.

### 2.5. MTT Assay

The MTT assay was used to estimate the metabolic activity of fibroblasts and their viability [33]. After a 24 h incubation of fibroblasts (1 × 10^4^ per well) in 96-well plates (SPL, Republic of Korea) with polymers and its composites with metals in a CO_2_-incubator (37°С, 5% CO_2_) manufactured by Thermo Fisher Scientific (USA). Briefly, 0.1 ml culture medium and 15 *μ*l MTT solution with the dye concentration of 5 mg/ml in PBS were added in each well for incubation. The fibroblasts were incubated with MTT in a CO_2_-incubator during 3 h at 37°С and 5% CO_2_. After collection of the medium, 0.1 ml DMSO with sodium dodecyl sulfate was added to dissolve formazan. The cells were incubated for 1 h at 37°С. The absorbance was measured at 570 nm. Cells treated for 5 min with 70% ice cold ethanol were used as a positive control. Quantitatively, the viability of fibroblasts was expressed as a percentage of viable cells compared with the controls and calculated in accordance with the following formula: (%) = [100 × (sample absorbance)/(control absorbance)].

### 2.6. Neutral Red Uptake Assay

In addition to MTT assay, the fibroblast viability was assessed by the neutral red uptake assay [34]. Briefly, the primary fibroblast culture cells were seeded in 96-well plates (1 × 10^4^ cells per well). They were incubated with the concentrations of composites described above during 24 h in a СО_2_-incubator. The neutral red dye solution (0.01%) in culture medium was added and the incubation lasted for 3 h in a СО_2_-incubator (5% CO_2_). The medium found in the samples was collected. Then, the dye was extracted and dissolved using 0.1 ml 50% ethanol and 3% acetic acid. After the dissolution of neutral red, the absorbance was measured at a wavelength of 570 nm. To quantify the viability degree, calculations were performed in accordance with the following formula: (%) = [100 × (sample absorbance)/(control absorbance)]. Numerical data were expressed as a percentage compared with the control samples.

### 2.7. Incubation with Blood

To assess the impact of polymer and hybrid metal-polymer composites on eryptosis indices, blood portions (50 *μ*l) were aliquoted from each sample to be incubated with various concentrations (0-0.1-0.2-0.5-1-2-5 mg/L) of pure D-PAA, Ag-D-PAA, and Au-D-PAA composites. Furthermore, the mixtures contained 5 ml RPMI-1640 medium with stable glutamine purchased from Biowest (France) and 5% fetal bovine serum purchased from BioWhittaker®, Lonza (Belgium) for 24 h. Thereafter, samples were used to obtain erythrocyte mass by double washing in phosphate buffer saline (PBS, pH 7.4, BD, Poland).

### 2.8. Annexin V Staining of Erythrocytes

Annexin V staining was used to assess phosphatidylserine exposure [35, 36]. According to the staining protocol, 2 *μ*l of erythrocyte mass was added to the freshly prepared 0.5 ml 1× Annexin-binding buffer purchased from BD Pharmingen™, BD Biosciences (San Jose, USA). To load the cells, 5 *μ*l fluorescein isothiocyanate- (FITC-) labeled Annexin V purchased from BD Pharmingen™, BD Biosciences (San Jose, USA) was dissolved in 100 *μ*l of the suspension described above. Loading was performed for 30 minutes in the dark. Directly prior to analysis of fluorescence by BD FACSCanto™ II Cell Analyzer (BD Biosciences, USA), 400 *μ*l 1× Annexin-binding buffer was added to each tube. Annexin V-FITC fluorescence was analyzed. The 488 nm laser was used for excitation, while the emitted light was collected at 520 nm. Per sample, 200,000 events were acquired. Postacquisition analysis was carried out by BD FACSDiva™ and FlowJo™ v10 software.

### 2.9. H2DCFDA Staining of Erythrocytes

ROS generation in erythrocytes treated with polymer and its hybrid composites with metals was analyzed by 2′,7′-dichlorodihydrofluorescein diacetate (H2DCFDA) staining [37]. The dye was purchased from Invitrogen™ (USA). Its 10 mM stock solution was prepared in dimethyl sulfoxide (DMSO) purchased from Sigma-Aldrich (USA) in advance and was stored at -20°C. Briefly, 2 *μ*l of erythrocyte mass was resuspended in PBS. The erythrocytes were loaded with the dye (5 *μ*M) and incubated for 30 min in the dark. Thereafter, the cells were washed twice and resuspended in 0.5 ml PBS. After a cascade of modifications in erythrocytes, H2DCFDA is converted to dichlorofluorescein (DCF) in cells under the influence of ROS. The DCF fluorescence was acquired at 520 nm after excitation with the 488 nm laser.

### 2.10. FLUO-4 AM Staining of Erythrocytes

Intraerythrocytic Ca^2+^ concentrations were assessed by FLUO4 AM staining. This Ca^2+^-sensitive dye was purchased from Becton Dickinson (USA). Its 5 mM stock solution was in anhydrous dimethyl sulfoxide (Sigma Aldrich, USA) that was prepared and used to stain 2 *μ*l of erythrocytes dissolved in 98 *μ*l PBS to obtain 2.5 *μ*M dye working solutions. The same volumes (400 *μ*l) of PBS were added after the incubation lasted for 30 min in the dark. The FLUO4 fluorescence was acquired at 520 nm after excitation with the 488 nm laser.

Fluorescence of Annexin V-FITC, DCF, and FLUO4 was expressed in arbitrary units (a.u.). Erythrocyte suspensions without the corresponding dyes were considered negative controls. Erythrocytes incubated with H_2_O_2_ (0.1 mM) were used as positive controls.

### 2.11. Statistics

Both for the experiments with fibroblasts and erythrocytes, the Kruskal-Wallis and *post hoc* Dunn's tests were used. Data are available as the median and interquartile range (IQR; 25–75%). *p* values not over 0.05 were interpreted as statistically significant. GraphPad Prism5.0 (GraphPad software, USA) was used to analyze data statistically.

## 3. Results

### 3.1. Characteristics of Metal/Polymer Composites

**Figure 1:**
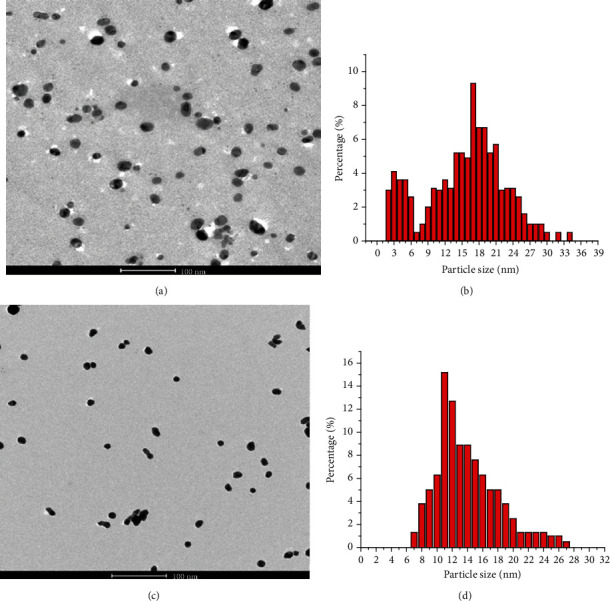
TEM images of Ag (a) and Au (c) nanoparticles into D-PAA matrix and the respective size distribution histograms (b, d).

The nanosystem characterization that was performed by using dynamic light scattering, transmission electron microscopy, and SAXS was referred in details in [20, 27–29]. Size distribution of nanoparticles is shown in Figure 1; (a, b) for Ag/D-PAA; (c, d) for Au/D-PAA.

All synthesized nanosystems were shown to be stable in time, and the size of Ag and Au nanoparticles was equal to 10-20 nm.

### 3.2. Characteristics of Fibroblast Cultures Exposed to Metal/Polymer Composites

**Figure 2:**
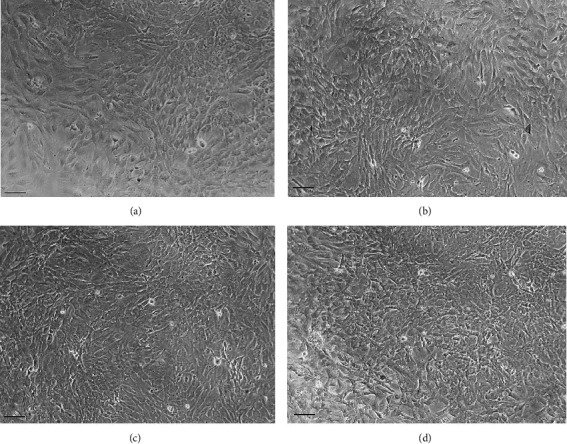
Representative images of fibroblast monolayers: intact (a), exposed to 50 mg/L star-shaped copolymer-nanocarriers with dextran core and grafted polyacrylamide chains (D-PAA) (b), 50 mg/L hybrid Ag/polymer composite (c), and 50 mg/L hybrid Au/polymer composite (d) for 24 h. Nanocomposites were found to have on significant impact on normal dermal fibroblast monolayers. The black scale bar is 100 *μ*m.

The cytotoxic effects of metal/D-PAA composites on fibroblasts were evaluated by analyzing their impact on fibroblast morphology, monolayer confluence, and cell adhesion. Microscopically, all the metal/polymer composites used in this study did not influence the cell shape and viability. The confluence of fibroblast monolayers was affected. The cells showed normal fibroblast morphology, and no cell debris was observed in the samples treated with 0.1-5 mg/L of various metal/D-PAA hybrid composites. Thus, fibroblasts were visually unaffected. Visually, no detached cells or small cell clusters were detected in the experimental samples suggesting that cell adhesion was unaffected in response to nanocomposites (Figure 2). Thus, our findings indicate that nanocomposites are nontoxic against fibroblasts at concentrations of 50 mg/L and below.

### 3.3. Cell Viability Assays

In addition, cytotoxicity profiles of nanocomposites were assessed by cell viability assays. Both cytotoxicity assays demonstrated that D-PAA, hybrid Ag/polymer, and hybrid Au/polymer composites did not reduce the survival of cells, evidenced by the absence of statistically significant changes in the optical density of formazan produced from MTT and neutral red culture media obtained from the fibroblast culture incubated with them for 24 h (see Table 1(s), supplementary files).

### 3.4. Eryptosis Indices

**Figure 3:**
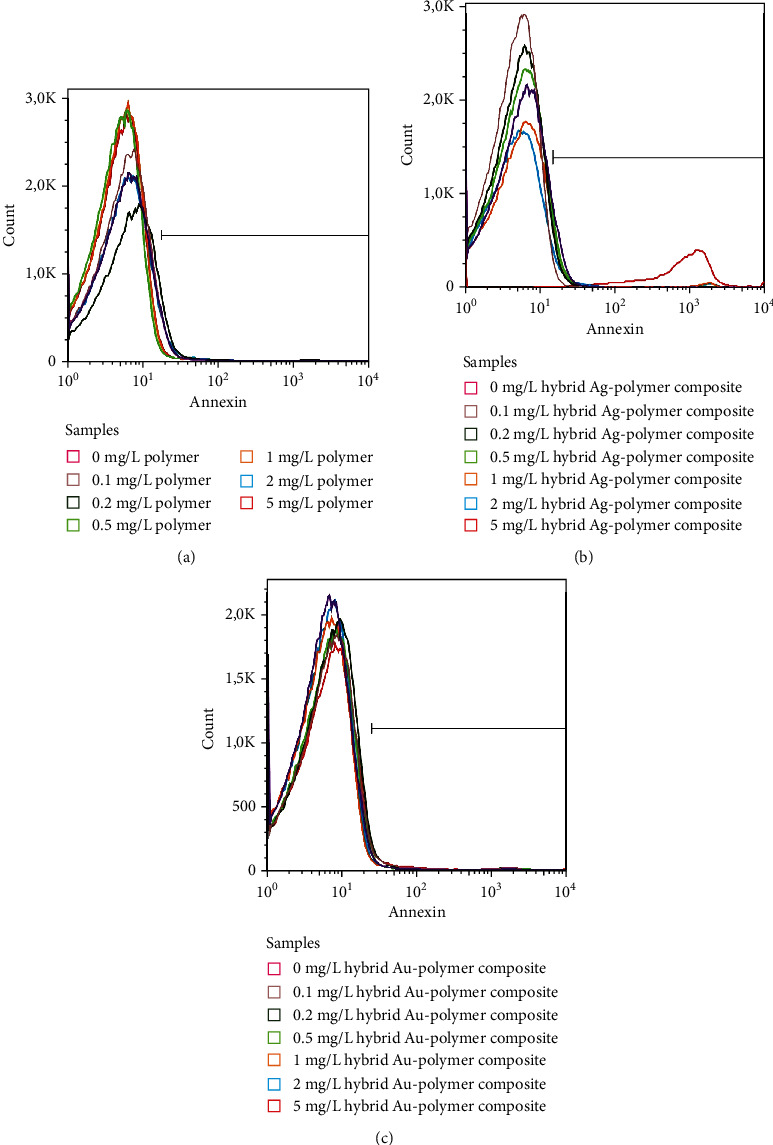
Representative histograms show the fluorescence of Annexin V-FITC in erythrocytes incubated with D-PAA (a), hybrid Ag/polymer composite (b), and hybrid Au/polymer composite (c) at concentrations of 0-0.1-0.2-0.5-1-2-5 mg/L for 24 h. The hybrid Ag-polymer composite was found to enhance Annexin V-FITC in erythrocytes at concentrations of over 5 mg/L suggesting induction of eryptosis.

Eryptosis rate and the role of ROS- and Ca^2+^-mediated mechanisms were analyzed by Annexin V-FITC, H2DCFDA, and FLUO4-AM staining, respectively. Phosphatidylserine exposure in cell membranes of erythrocytes exposed to nanocomposites for 24 h was characterized by comparing the percentage of Annexin V-positive cells and MFI values of Annexin V-FITC. These eryptosis indices were unaffected in cells treated with D-PAA and hybrid Au-polymer composite at any concentration used, while hybrid Ag/polymer composite at a concentration of 5 mg/L increased both the amount of Annexin V-displaying cells and MFI values of Annexin V-FITC (Figure 3, Table 2 s supplementary files). This indicates activation of eryptosis in response to hybrid Ag/polymer composite.

**Figure 4:**
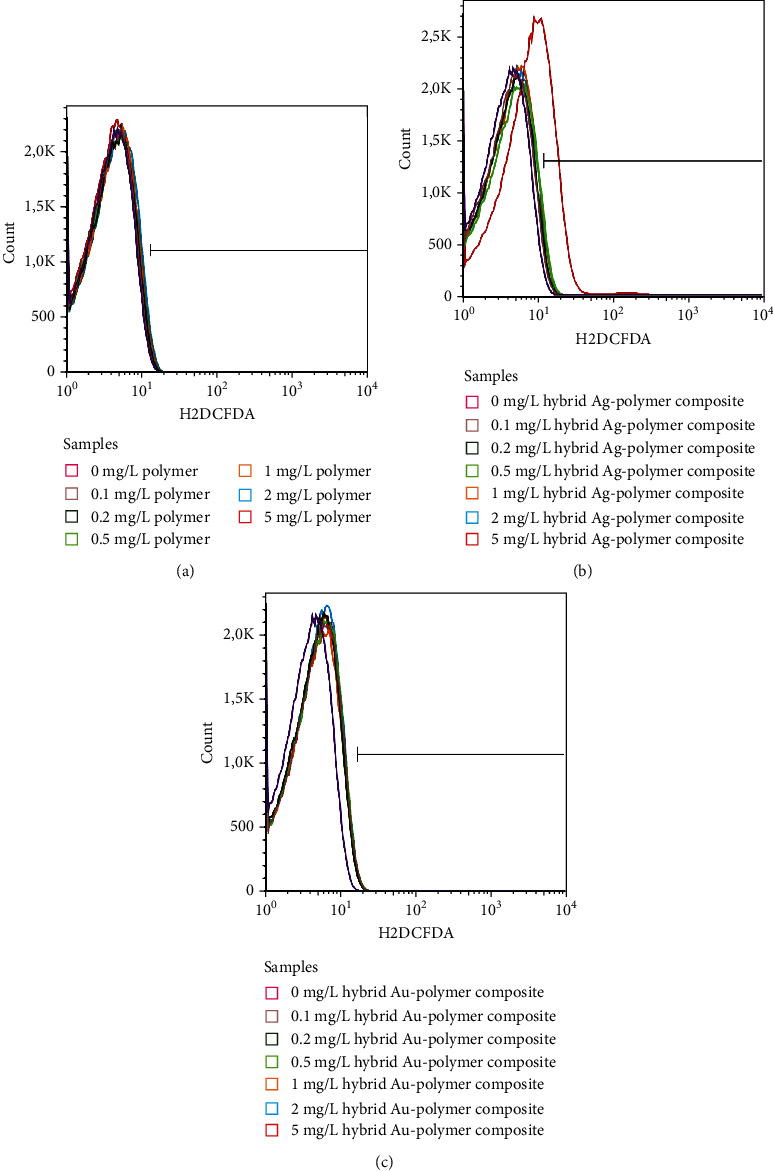
Representative histograms show the fluorescence of dichlorofluorescein in erythrocytes exposed to D-PAA (a), hybrid Ag/polymer composite (b), and hybrid Au/polymer composite (c) at concentrations 0-0.1-0.2-0.5-1-2-5 mg/L for 24 h. Dichlorofluorescein fluorescence is dependent on intracellular reactive oxygen species (ROS) levels. The hybrid Ag/D-PAA composite was revealed to induce ROS generation in erythrocytes at concentrations above 5 mg/L.

H2DCFDA staining revealed that D-PAA and hybrid A/D-PAA composite did not increase the fluorescence of DCF in erythrocytes, which indicates the absence of stimulatory effects on ROS generation. In contrast to these materials, Ag/polymer composite statistically significantly increased MFI values of DCF (*p* < 0.0001) when used at a concentration of 5 mg/L, while lower concentrations had no influence of ROS production (Figure 4, Table 2 s supplementary files).

**Figure 5:**
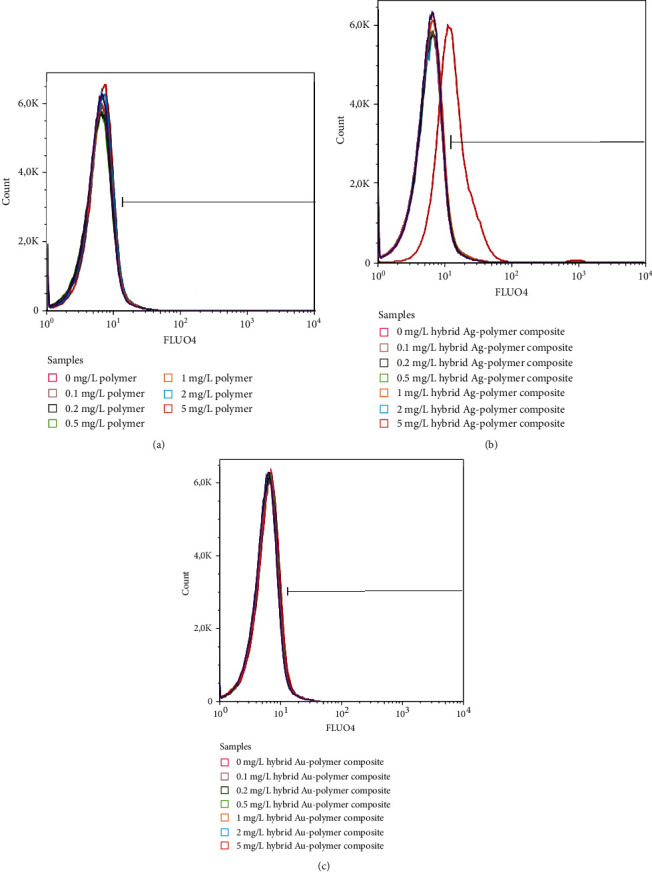
Representative histograms allow comparing the fluorescence of Ca^2+^-sensitive FLUO4 dye in erythrocytes treated with D-PAA (a), hybrid Ag/polymer composite (b), and hybrid Au/polymer composite (c) at concentrations 0-0.1-0.2-0.5-1-2-5 mg/L during 24 h. The highest concentration (5 mg/L) of hybrid Ag/D-PAA composite increases intracellular calcium ion levels in erythrocytes.

FLUO4-AM staining allowed comparing the effects of various composites on intracellular Ca^2+^levels. Two parameters of FLUO4-AM staining such as the amount of cells with high FLUO4 fluorescence and MFI values of FLUO4 were calculated and compared. Both Ca^2 +^ -characterizing parameters were not changed in erythrocytes incubated with D-PAA and Au-containing composite. Meanwhile, in erythrocytes exposed to 5 mg/L of Ag-containing composite, both indices were statistically significantly elevated (*p* < 0.0001) pointing to an increase in intraerythrocytic calcium ion levels (Figure 5, Table 2 s supplementary files).

## 4. Discussion

Nanomaterials have drawn a lot of attention as novel diagnostic and therapeutic tools. However, despite recent advancements in biomedical research, the bench-to-bedside transition is still below the desired level. Only a few dozen nanoparticles are under the clinical trials at the moment [38]. Implementation of most nanoparticles in the clinical practice is limited in most cases due to their toxicity. Thus, nanosafety studies are of huge importance to ensure adequate evaluation of nanotoxicity to prevent detrimental effects of nanomaterials on the human health and the environment. Since the toxicity remains one of the major challenges in nanomedicine, the development of standardized and widely recognized protocols to introduce and maintain the high quality of nanosafety testing is of exceptional importance [39].

Viability assays performed on fibroblast cell lines remain a simple, physiologically relevant and widely used tool to assess the nanotoxicity *in vitro* [40]. Our findings indicate that D-PAA itself and its hybrid composites with Ag and Au did not reduce the viability of normal dermal rat fibroblasts at concentrations of at least up to 50 mg/L. Given the well-known toxicity of noble metals, our findings supplement experimental data on the hypothesis that coating reduces their cytotoxicity [41].

The search for novel, more sensitive and effective approaches to assess the biocompatibility of nanosized materials has revealed that eryptosis is a promising model in nanotoxicology [42, 43].

Eryptosis is a suicidal cell death of erythrocytes associated with cell shrinkage, membrane blebbing, and phospholipid scrambling on the plasma membrane, triggered under unfavorable circumstance such as energy deficit, hyperosmolarity, exposure to xenobiotics, calcium ion entry, ceramide accumulation, activation of caspases and calpain, and imbalance of redox homeostasis [44]. Eryptosis aims at removing dysfunctional erythrocytes from the bloodstream, which occurs by macrophages by efferocytosis [45]. There is a growing body of evidence that phosphatidylserine translocation from the inner membrane leaflet to the outer one, ROS overgeneration and Ca^2+^ entry are the hallmarks of eryptosis [46, 47]. In this study, five eryptosis indices, which reflect the processes mentioned above, were determined in erythrocytes exposed to varying concentrations (0-5 mg/L) of metal/D-PAA composites. Our findings suggest that eryptosis is induced by hybrid Ag-polymer composite at the concentration of 5 mg/L only, evidenced by the increased phosphatidylserine exposure. Expectedly, these eryptotic erythrocytes have higher cytosolic calcium ion levels and excessive ROS production indicating the role of ROS- and Ca^2+^-mediated pathways in hybrid Ag-polymer composite-induced eryptosis. Silver nanoparticles are known to induce ROS generation and promote oxidative stress [48, 49]. It can be assumed that Ag-induced oxidative stress stimulated Ca^2+^ influx. In its turn, calcium ions activate calpains, a family of cysteine proteolytic enzymes, responsible for degradation of erythrocyte cytoskeleton [50]. However, the detailed molecular mechanisms that mediate noble-metal-polymer composites-mediated eryptosis should be elucidated in further studies. Our findings are consistent with the reports on the ability of polyvinylpyrrolidone- and citrate-coated Ag nanoparticles to induce eryptosis involving ROS- and Ca^2+^-mediated pathways [42]. In addition, calpain activation was required. Notably, nanoparticles triggered eryptosis at a lower concentration compared with the current experiment. In another study, polyethylene glycol coated Au nanorods were found not to induce eryptosis. However, cetyltrimethylammonium bromide-coated Au nanorods were experimentally shown to trigger eryptosis in a Ca^2+^- and ROS-dependent manner. Of note, the authors attribute the proeryptotic effects to coating [51].

It can be stated that Au/polymer composite is less cytotoxic than a composite with Ag. These findings are consistent with the experimental data on the comparison of Ag and Au nanoparticle toxicity [52, 53]. Thus, incorporation of noble metal in the polymer does not alter this regularity.

Eryptosis is a more sensitive tool for assessing the biosafety profile of nanosized materials compared with fibroblast viability assays. Of note, its sensitivity is higher than that of viability assays on cultured fibroblasts not only in case of noble metal-polymer hybrid composites, but for different TiO_2_ nanoparticles as well [54].

## 5. Conclusions

Our findings suggest that metal/D-PAA composites show no cytotoxicity at low concentrations. Ag/polymer composite is more toxic compared to D-PAA and hybrid Au/D-PAA composite. Thus, they are promising biocompatible agents that can be used for medical purposes, partially as a biocompatible therapeutic effect-enhancing component in the multicomponent nanosystems. The study provides experimental evidence that eryptosis is a sensitive approach to assess the biocompatibility of nanomaterials.

## Figures and Tables

**Table 1:** Molecular parameters of D-PAA copolymer.

Polymer	*M*_*w*_ × 10^−6^ g/mol	*R*_*g*_, nm	*M*_*w*_/*M*_*n*_
D-PAA	2.15	85	1.72

M_w_: weight average molecular weight; R_g_: radius of gyration; and M_w_/M_n_: polydispersity index.

## Data Availability

The data that support the findings of this study are available from the corresponding author, Guochao Nie, upon reasonable request.

## Supplementary Materials

Supplementary Materials Table 1S: cell viability assays performed on cultured fibroblasts exposed to D-PAA, hybrid Ag/polymer composite, and hybrid Au/polymer composite. Note: D-PAA: dextran-graft-polyacrylamide. Table 2S: eryptosis parameters in red blood cells exposed to D-PAA, hybrid Ag/polymer composite, and hybrid Au/polymer composite. Note: D-PAA: dextran-graft-polyacrylamide; DCF: dichlorofluorescein; FITC: fluorescein isothiocyanate; MFI: mean fluorescence intensity.
