# Supplementary material for: Cytotoxicity of Hybrid Noble Metal-Polymer Composites
Source: Biomed Res Int. 2022 Oct 11;2022:1487024. doi: 10.1155/2022/1487024 (PMC9578826; doi:10.1155/2022/1487024)
Supplement: Supplementary Materials — Table 1S: cell viability assays performed on cultured fibroblasts exposed to D-PAA, hybrid Ag/polymer composite, and hybrid Au/polymer composite. Note: D-PAA: dextran-graft-polyacrylamide. Table 2S: eryptosis parameters in red blood cells exposed to D-PAA, hybrid Ag/polymer composite, and hybrid Au/polymer composite. Note: D-PAA: dextran-graft-polyacrylamide; DCF: dichlorofluorescein; FITC: fluorescein isothiocyanate; MFI: mean fluorescence intensity. [file 1487024.f1.zip › Supplementary table 1.docx]

**Supplementary file**

Table 1S

**Cell viability assays performed on cultured fibroblasts exposed to D-PAA, hybrid Ag/polymer composite and hybrid Au/polymer composite**

| Eryptosis index | Concentrations | D-PAA | Hybrid Ag/polymer composite | Hybrid Au/polymer composite |
| --- | --- | --- | --- | --- |
| MTT assay, viable cells compared to control samples, % | 2 mg / L | 101.1 [100.3; 107.0] %, p > 0.05 | 81.1 [69.0; 93.5] %, p > 0.05 | 103.1 [98.0; 111.6] %, p > 0.05 |
|  | 5 mg / L | 100.7 [96.6; 103.7] %, p > 0.05 | 80.8 [70.1; 84.8] %, p > 0.05 | 103.4 [98.6; 109.3] %, p > 0.05 |
|  | 10 mg / L | 103.1 [100.9; 10.6] %, p > 0.05 | 81.9 [72.9; 88.5] %, p > 0.05 | 104.7 [98.9; 109.5] %, p > 0.05 |
|  | 20 mg / L | 102.4 [95.9; 104.1] %, p > 0.05 | 81.4 [75.0; 84.5] %, p > 0.05 | 104.9 [98.3; 112.5] %, p > 0.05 |
|  | 30 mg / L | 102.8 [98.9; 106.5] %, p > 0.05 | 84.0 [81.9; 92.8] %, p > 0.05 | 98.5 [92.7; 104.9] %, p > 0.05 |
|  | 50 mg / L | 106.4 [102.1; 110.4] %, p > 0.05 | 94.6 [88.7; 104.0] %, p > 0.05 | 111.9 [108.1; 1130] %, p > 0.05 |
| Neutral red uptake assay, viable cells compared to control samples, % | 2 mg / L | 114.2 [109.5; 121.8] %, p > 0.05 | 93.6 [86.9; 101.7] %, p > 0.05 | 104.5 [84.2; 119.8] %, p > 0.05 |
|  | 5 mg / L | 94.6 [88.7; 120.4] %, p > 0.05 | 102.4 [91.4; 118.0] %, p > 0.05 | 98.7 [87.9; 129.3] %, p > 0.05 |
|  | 10 mg / L | 102.6 [90.2; 117.8] %, p > 0.05 | 91.1 [83.0; 108.7] %, p > 0.05 | 105.3 [93.8; 115.3] %, p > 0.05 |
|  | 20 mg / L | 109.8 [98.3; 123.9] %, p > 0.05 | 89.4 [75.3; 93.9] %, p > 0.05 | 110.9 [90.0; 121.3] %, p > 0.05 |
|  | 30 mg / L | 121.6 [100.6; 133.3] %, p > 0.05 | 101.1 [90.6; 110.4] %, p > 0.05 | 97.7 [91.5; 108.6] %, p > 0.05 |
|  | 50 mg / L | 81.5 [77.3; 97.2] %, p > 0.05 | 95.3 [80.9; 96.1] %, p > 0.05 | 96.0 [87.3; 127.0] %, p > 0.05 |

**Note:** D-PAA– dextran-graft-polyacrylamide.
