# Supplementary material for: Cytotoxicity of Hybrid Noble Metal-Polymer Composites
Source: Biomed Res Int. 2022 Oct 11;2022:1487024. doi: 10.1155/2022/1487024 (PMC9578826; doi:10.1155/2022/1487024)
Supplement: Supplementary Materials — Table 1S: cell viability assays performed on cultured fibroblasts exposed to D-PAA, hybrid Ag/polymer composite, and hybrid Au/polymer composite. Note: D-PAA: dextran-graft-polyacrylamide. Table 2S: eryptosis parameters in red blood cells exposed to D-PAA, hybrid Ag/polymer composite, and hybrid Au/polymer composite. Note: D-PAA: dextran-graft-polyacrylamide; DCF: dichlorofluorescein; FITC: fluorescein isothiocyanate; MFI: mean fluorescence intensity. [file 1487024.f1.zip › Supplementary table 2.docx]

**Supplementary file**

Table 2S

**Eryptosis parameters in red blood cells exposed to D-PAA, hybrid Ag/polymer composite and hybrid Au/polymer composite**

| Eryptosis index | Concentrations | D-PAA | Hybrid Ag/polymer composite | Hybrid Au/polymer composite |
| --- | --- | --- | --- | --- |
| Amount of annexin V-positive cells, % | 0 mg / L | 1.15 [0.98; 2.65] % | 1.15 [0.98; 2.65] % | 1.15 [0.98; 2.65] % |
|  | 0.1 mg / L | 1.80 [0.85; 2.30] %, p > 0.05 | 1.20 [0.73; 2.00] %, p > 0.05 | 2.20 [1.63; 3.43] %, p > 0.05 |
|  | 0.2 mg / L | 2.00 [1.13; 2.73] %, p > 0.05 | 1.55 [1.25; 2.20] %, p > 0.05 | 3.00 [1.85; 3.73] %, p > 0.05 |
|  | 0.5 mg / L | 1.25 [0.88; 2.48] %, p > 0.05 | 1.90 [1.05; 2.33] %, p > 0.05 | 2.15 [1.15; 2.98] %, p > 0.05 |
|  | 1 mg / L | 1.80 [1.25; 2.18] %, p > 0.05 | 2.80 [2.40; 3.33] %, p > 0.05 | 2.50 [1.13; 4.18] %, p > 0.05 |
|  | 2 mg / L | 2.10 [1.18; 2.83] %, p > 0.05 | 4.00 [2.88; 4.83] %, p > 0.05 | 2.40 [1.90; 3.45] %, p > 0.05 |
|  | 5 mg / L | 1.65 [0.88; 2.18] %, p > 0.05 | 10.55 [9.15; 12.08] %, p < 0.0001 | 2.90 [2.35; 3.88] %, p > 0.05 |
| MFI of annexin V-FITC, a.u. | 0 mg / L | 187 [172; 268] a.u. | 187 [172; 268] a.u. | 187 [172; 268] a.u. |
|  | 0.1 mg / L | 180 [166; 203] a.u., p > 0.05 | 167 [144; 194] a.u., p > 0.05 | 223 [186; 282] a.u., p > 0.05 |
|  | 0.2 mg / L | 210 [181; 282] a.u., p > 0.05 | 264 [230; 304] a.u., p > 0.05 | 267 [231; 307] a.u., p > 0.05 |
|  | 0.5 mg / L | 190 [165; 233] a.u., p > 0.05 | 292 [219; 381] a.u., p > 0.05 | 292 [254; 320] a.u., p > 0.05 |
|  | 1 mg / L | 232 [176; 292] a.u., p > 0.05 | 401 [339; 486] a.u., p > 0.05 | 266 [201; 398] a.u., p > 0.05 |
|  | 2 mg / L | 268 [240; 293] a.u., p > 0.05 | 425 [363; 492] a.u., p > 0.05 | 291 [228; 309] a.u., p > 0.05 |
|  | 5 mg / L | 229 [188; 311] a.u., p > 0.05 | 897 [771; 1309] a.u., p < 0.0001 | 322 [274; 379] a.u., p > 0.05 |
| MFI of DCF, a.u. | 0 mg / L | 66 [49; 86] a.u. | 66 [49; 86] a.u. | 66 [49; 86] a.u. |
|  | 0.1 mg / L | 66 [55; 77] a.u., p > 0.05 | 70 [51; 80] a.u., p > 0.05 | 57 [49; 63] a.u., p > 0.05 |
|  | 0.2 mg / L | 61 [51; 75] a.u., p > 0.05 | 80 [61; 92] a.u., p > 0.05 | 60 [53; 79] a.u., p > 0.05 |
|  | 0.5 mg / L | 74 [54; 84] a.u., p > 0.05 | 68 [53; 78] a.u., p > 0.05 | 66 [58; 74] a.u., p > 0.05 |
|  | 1 mg / L | 82 [55; 88] a.u., p > 0.05 | 69 [64; 78] a.u., p > 0.05 | 55 [48; 80] a.u., p > 0.05 |
|  | 2 mg / L | 73 [55; 88] a.u., p > 0.05 | 80 [72; 92] a.u., p > 0.05 | 63 [56; 69] a.u., p > 0.05 |
|  | 5 mg / L | 75 [54; 99] a.u., p > 0.05 | 147 [130; 153] a.u., p = 0.0061 | 63 [56; 71] a.u., p > 0.05 |
| Amount of cells with high fluorescence of FLUO4, % | 0 mg / L | 2.15 [1.88; 3.05] % | 2.15 [1.88; 3.05] % | 2.15 [1.88; 3.05] % |
|  | 0.1 mg / L | 2.35 [1.80; 3.33] %, p > 0.05 | 2.40 [1.98; 3.43] %, p > 0.05 | 2.15 [1.88; 2.70] %, p > 0.05 |
|  | 0.2 mg / L | 2.55 [1.93; 2.98] %, p > 0.05 | 2.70 [2.05; 3.65] %, p > 0.05 | 2.60 [2.00; 4.18] %, p > 0.05 |
|  | 0.5 mg / L | 2.30 [1.93; 3.48] %, p > 0.05 | 2.20 [2.05; 2.63] %, p > 0.05 | 2.45 [1.85; 3.20] %, p > 0.05 |
|  | 1 mg / L | 2.55 [1.93; 2.93] %, p > 0.05 | 2.45 [1.98; 2.75] %, p > 0.05 | 2.60 [1.80; 3.50] %, p > 0.05 |
|  | 2 mg / L | 2.30 [1.68; 2.68] %, p > 0.05 | 5.30 [4.03; 6.43] %, p > 0.05 | 2.25 [1.63; 4.05] %, p > 0.05 |
|  | 5 mg / L | 2.55 [2.33; 3.08] %, p > 0.05 | 17.80 [13.53; 24.98] %, p < 0.0001 | 2.85 [2.18; 3.50] %, p > 0.05 |
| MFI of FLUO4, a.u. | 0 mg / L | 178 [170; 211] a.u. | 178 [170; 211] a.u. | 178 [170; 211] a.u. |
|  | 0.1 mg / L | 201 [168; 224] a.u., p > 0.05 | 179 [165; 218] a.u., p > 0.05 | 192 [178; 247] a.u., p > 0.05 |
|  | 0.2 mg / L | 187 [174; 210] a.u., p > 0.05 | 205 [171; 284] a.u., p > 0.05 | 189 [176; 200] a.u., p > 0.05 |
|  | 0.5 mg / L | 194 [184; 240] a.u., p > 0.05 | 191 [176; 196] a.u., p > 0.05 | 195 [181; 212] a.u., p > 0.05 |
|  | 1 mg / L | 210 [193; 266] a.u., p > 0.05 | 220 [181; 284] a.u., p > 0.05 | 194 [182; 201] a.u., p > 0.05 |
|  | 2 mg / L | 197 [177; 230] a.u., p > 0.05 | 221 [187; 296] a.u., p > 0.05 | 195 [183; 204] a.u., p > 0.05 |
|  | 5 mg / L | 222 [192; 302] a.u., p > 0.05 | 482 [374; 535] a.u., p < 0.0001 | 194 [186; 216] a.u., p > 0.05 |

**Note:** D-PAA– dextran-graft-polyacrylamide; DCF – dichlorofluorescein; FITC – fluorescein isothiocyanate; MFI – mean fluorescence intensity
